# Experimental exploration of five-qubit quantum error-correcting code with superconducting qubits

**DOI:** 10.1093/nsr/nwab011

**Published:** 2021-01-21

**Authors:** Ming Gong, Xiao Yuan, Shiyu Wang, Yulin Wu, Youwei Zhao, Chen Zha, Shaowei Li, Zhen Zhang, Qi Zhao, Yunchao Liu, Futian Liang, Jin Lin, Yu Xu, Hui Deng, Hao Rong, He Lu, Simon C Benjamin, Cheng-Zhi Peng, Xiongfeng Ma, Yu-Ao Chen, Xiaobo Zhu, Jian-Wei Pan

**Affiliations:** Hefei National Laboratory for Physical Sciences at the Microscale and Department of Modern Physics, University of Science and Technology of China, Hefei 230026, China; Shanghai Branch, CAS Center for Excellence and Synergetic Innovation Center in Quantum Information and Quantum Physics, University of Science and Technology of China, Shanghai 201315, China; Shanghai Research Center for Quantum Sciences, Shanghai 201315, China; Hefei National Laboratory for Physical Sciences at the Microscale and Department of Modern Physics, University of Science and Technology of China, Hefei 230026, China; Shanghai Branch, CAS Center for Excellence and Synergetic Innovation Center in Quantum Information and Quantum Physics, University of Science and Technology of China, Shanghai 201315, China; Center for Quantum Information, Institute for Interdisciplinary Information Sciences, Tsinghua University, Beijing 100084, China; Department of Materials, University of Oxford, Oxford OX1 3PH, UK; Hefei National Laboratory for Physical Sciences at the Microscale and Department of Modern Physics, University of Science and Technology of China, Hefei 230026, China; Shanghai Branch, CAS Center for Excellence and Synergetic Innovation Center in Quantum Information and Quantum Physics, University of Science and Technology of China, Shanghai 201315, China; Shanghai Research Center for Quantum Sciences, Shanghai 201315, China; Hefei National Laboratory for Physical Sciences at the Microscale and Department of Modern Physics, University of Science and Technology of China, Hefei 230026, China; Shanghai Branch, CAS Center for Excellence and Synergetic Innovation Center in Quantum Information and Quantum Physics, University of Science and Technology of China, Shanghai 201315, China; Shanghai Research Center for Quantum Sciences, Shanghai 201315, China; Hefei National Laboratory for Physical Sciences at the Microscale and Department of Modern Physics, University of Science and Technology of China, Hefei 230026, China; Shanghai Branch, CAS Center for Excellence and Synergetic Innovation Center in Quantum Information and Quantum Physics, University of Science and Technology of China, Shanghai 201315, China; Shanghai Research Center for Quantum Sciences, Shanghai 201315, China; Hefei National Laboratory for Physical Sciences at the Microscale and Department of Modern Physics, University of Science and Technology of China, Hefei 230026, China; Shanghai Branch, CAS Center for Excellence and Synergetic Innovation Center in Quantum Information and Quantum Physics, University of Science and Technology of China, Shanghai 201315, China; Shanghai Research Center for Quantum Sciences, Shanghai 201315, China; Hefei National Laboratory for Physical Sciences at the Microscale and Department of Modern Physics, University of Science and Technology of China, Hefei 230026, China; Shanghai Branch, CAS Center for Excellence and Synergetic Innovation Center in Quantum Information and Quantum Physics, University of Science and Technology of China, Shanghai 201315, China; Shanghai Research Center for Quantum Sciences, Shanghai 201315, China; Center for Quantum Information, Institute for Interdisciplinary Information Sciences, Tsinghua University, Beijing 100084, China; Center for Quantum Information, Institute for Interdisciplinary Information Sciences, Tsinghua University, Beijing 100084, China; Center for Quantum Information, Institute for Interdisciplinary Information Sciences, Tsinghua University, Beijing 100084, China; Hefei National Laboratory for Physical Sciences at the Microscale and Department of Modern Physics, University of Science and Technology of China, Hefei 230026, China; Shanghai Branch, CAS Center for Excellence and Synergetic Innovation Center in Quantum Information and Quantum Physics, University of Science and Technology of China, Shanghai 201315, China; Shanghai Research Center for Quantum Sciences, Shanghai 201315, China; Hefei National Laboratory for Physical Sciences at the Microscale and Department of Modern Physics, University of Science and Technology of China, Hefei 230026, China; Shanghai Branch, CAS Center for Excellence and Synergetic Innovation Center in Quantum Information and Quantum Physics, University of Science and Technology of China, Shanghai 201315, China; Shanghai Research Center for Quantum Sciences, Shanghai 201315, China; Hefei National Laboratory for Physical Sciences at the Microscale and Department of Modern Physics, University of Science and Technology of China, Hefei 230026, China; Shanghai Branch, CAS Center for Excellence and Synergetic Innovation Center in Quantum Information and Quantum Physics, University of Science and Technology of China, Shanghai 201315, China; Shanghai Research Center for Quantum Sciences, Shanghai 201315, China; Hefei National Laboratory for Physical Sciences at the Microscale and Department of Modern Physics, University of Science and Technology of China, Hefei 230026, China; Shanghai Branch, CAS Center for Excellence and Synergetic Innovation Center in Quantum Information and Quantum Physics, University of Science and Technology of China, Shanghai 201315, China; Shanghai Research Center for Quantum Sciences, Shanghai 201315, China; Hefei National Laboratory for Physical Sciences at the Microscale and Department of Modern Physics, University of Science and Technology of China, Hefei 230026, China; Shanghai Branch, CAS Center for Excellence and Synergetic Innovation Center in Quantum Information and Quantum Physics, University of Science and Technology of China, Shanghai 201315, China; Shanghai Research Center for Quantum Sciences, Shanghai 201315, China; Hefei National Laboratory for Physical Sciences at the Microscale and Department of Modern Physics, University of Science and Technology of China, Hefei 230026, China; Shanghai Branch, CAS Center for Excellence and Synergetic Innovation Center in Quantum Information and Quantum Physics, University of Science and Technology of China, Shanghai 201315, China; Department of Materials, University of Oxford, Oxford OX1 3PH, UK; Hefei National Laboratory for Physical Sciences at the Microscale and Department of Modern Physics, University of Science and Technology of China, Hefei 230026, China; Shanghai Branch, CAS Center for Excellence and Synergetic Innovation Center in Quantum Information and Quantum Physics, University of Science and Technology of China, Shanghai 201315, China; Shanghai Research Center for Quantum Sciences, Shanghai 201315, China; Center for Quantum Information, Institute for Interdisciplinary Information Sciences, Tsinghua University, Beijing 100084, China; Hefei National Laboratory for Physical Sciences at the Microscale and Department of Modern Physics, University of Science and Technology of China, Hefei 230026, China; Shanghai Branch, CAS Center for Excellence and Synergetic Innovation Center in Quantum Information and Quantum Physics, University of Science and Technology of China, Shanghai 201315, China; Shanghai Research Center for Quantum Sciences, Shanghai 201315, China; Hefei National Laboratory for Physical Sciences at the Microscale and Department of Modern Physics, University of Science and Technology of China, Hefei 230026, China; Shanghai Branch, CAS Center for Excellence and Synergetic Innovation Center in Quantum Information and Quantum Physics, University of Science and Technology of China, Shanghai 201315, China; Shanghai Research Center for Quantum Sciences, Shanghai 201315, China; Hefei National Laboratory for Physical Sciences at the Microscale and Department of Modern Physics, University of Science and Technology of China, Hefei 230026, China; Shanghai Branch, CAS Center for Excellence and Synergetic Innovation Center in Quantum Information and Quantum Physics, University of Science and Technology of China, Shanghai 201315, China; Shanghai Research Center for Quantum Sciences, Shanghai 201315, China

**Keywords:** quantum error-correcting code, superconducting qubit, five-qubit code, error detection, logical operation

## Abstract

Quantum error correction is an essential ingredient for universal quantum computing. Despite tremendous experimental efforts in the study of quantum error correction, to date, there has been no demonstration in the realisation of universal quantum error-correcting code, with the subsequent verification of all key features including the identification of an arbitrary physical error, the capability for transversal manipulation of the logical state and state decoding. To address this challenge, we experimentally realise the [5, 1, 3] code, the so-called smallest perfect code that permits corrections of generic single-qubit errors. In the experiment, having optimised the encoding circuit, we employ an array of superconducting qubits to realise the [5, 1, 3] code for several typical logical states including the magic state, an indispensable resource for realising non-Clifford gates. The encoded states are prepared with an average fidelity of $57.1(3)\%$ while with a high fidelity of $98.6(1)\%$ in the code space. Then, the arbitrary single-qubit errors introduced manually are identified by measuring the stabilisers. We further implement logical Pauli operations with a fidelity of $97.2(2)\%$ within the code space. Finally, we realise the decoding circuit and recover the input state with an overall fidelity of $74.5(6)\%$, in total with 92 gates. Our work demonstrates each key aspect of the [5, 1, 3] code and verifies the viability of experimental realisation of quantum error-correcting codes with superconducting qubits.

## Teaser

Experimental demonstration of encoding, decoding, logical operation, and error detection of a five-qubit error correcting code on a superconducting quantum processor.

## INTRODUCTION

Quantum computers can tackle classically intractable problems [1] and efficiently simulate many-body quantum systems [2]. However, quantum computers are notoriously difficult to control, due to their ubiquitous yet inevitable interaction with their environment, together with imperfect manipulations that constitute the algorithm. The theory of fault tolerance has been developed as the long-term solution to this issue, enabling universal error-free quantum computing with noisy quantum hardware [3–7]. The logical qubits of an algorithm can be represented using a larger number of flawed physical qubits. Providing that the machine is sufficiently large (high qubit count), and that physical errors happen with a probability below a certain threshold, then such errors can be systematically detected and corrected [8,9]. In experiment, several small quantum error-correcting codes (QECCs), including the repetition code [10–16], the four-qubit error-detecting code [17–19], the seven-qubit color code [20], the bosonic quantum error-correcting code [21,22] and others [23–26], have been realised with different hardware platforms. These works have shown the success of realising error-correcting codes with non-destructive stabiliser measurements and their application in extending the system lifetime [19,25]. Nevertheless, previous experiments are limited to restricted codes for correcting certain types of errors or the preparation of specific logical states. It remains an open challenge to realise a fully-functional QECC.

Here, we focus on the five-qubit [5, 1, 3] code, the ‘perfect’ code that can protect a logical qubit from an arbitrary single physical error using the smallest number of qubits [6,7]. While proof-of-principle experimental demonstrations of the [5, 1, 3] code have been conducted on NMR systems [27], whether it could be incorporated in more scalable quantum computing systems and protect errors presented in these systems remain open. Here, we focus on the realisation of the five-qubit code with superconducting qubit systems. As a preparatory theoretical step, we recompile the universal encoding circuit that prepares an arbitrary logical state in order to realise it with the fewest possible number of nearest-neighbour two-qubit gates. In experiment, we implement basic functions of the code by realising logical state preparation, transversal logical operations and state decoding.

## THEORY

The five-qubit [5, 1, 3] code is a type of stabiliser code that is defined by a set of independent operators from the Pauli group, called stabilisers, such that the code space only has eigenvalue +1. The four stabilisers of the five-qubit code are

(1) $$\begin
{eqnarray}
g_1 &=& X_1Z_2Z_3X_4I_5, \quad g_2 = I_1X_2Z_3Z_4X_5, \nonumber\\
g_3 &=& X_1I_2X_3Z_4Z_5, \quad g_4 = Z_1X_2I_3X_4Z_5, \nonumber\\
\end{eqnarray}$$

with *I*_*i*_, *X*_*i*_, *Y*_*i*_, *Z*_*i*_ being the Pauli matrices acting on the *i*th qubit. The logical state space is defined by states $\mathinner {|{\Psi }\rangle }_L=a\mathinner {|{0}\rangle }_L+b\mathinner {|{1}\rangle }_L$ that are simultaneously stabilised by the four stabilisers with $g_i\mathinner {|{\Psi }\rangle }_L = +\mathinner {|{\Psi }\rangle }_L$ for all *i* = 1, 2, 3, 4. Here, the logical states $\mathinner {|{0}\rangle }_L$ and $\mathinner {|{1}\rangle }_L$ are the basis states that are eigenstates of the logical *Z*_*L*_ operator. Any logical state $\mathinner {|{\Psi }\rangle }_L$ can be uniquely determined by the four stabilisers defined in Equation (1) together with the fifth stabiliser $g_5=\mathinner {|{\Psi }\rangle }\mathinner {\langle {\Psi }|}_L-\mathinner {|{\Psi ^\bot }\rangle }\mathinner {\langle {\Psi ^\bot }|}_L =(aa^\star -bb^\star ){Z}_L+(a^\star b\,+\,b^\star a){X}_L\,-\,i(a^\star b\,-\,b^\star a){Y}_L$, with $\mathinner {|{\Psi ^\bot }\rangle }=b^\star \mathinner {|{0_L}\rangle }-a^\star \mathinner {|{1_L}\rangle }$. That is, any logical state $\mathinner {|{\Psi }\rangle }_L$ can be decomposed as $\mathinner {|{\Psi }\rangle }_L\mathinner {\langle {\Psi }|}_L =2^{-5}\prod _{i=1}^5(g_0+g_i)$, where *g*_0_ = *I*_1_*I*_2_*I*_3_*I*_4_*I*_5_ is the trivial stabiliser of all pure quantum states. Logical Pauli operators are transversely realised by applying the corresponding single-qubit gates on each physical qubit, σ_*L*_ = σ_1_σ_2_σ_3_σ_4_σ_5_ for σ = *X*, *Y*, *Z*. General logical operators, such as the $T_L=e^{-iZ_L\pi /8}$ gate, may not be transversely realised.

The five-qubit code has distance three and therefore all single-qubit errors can be identified (and thus corrected) while all double-qubit errors can be detected. When there is no error, all stabiliser measurements should yield +1 for the encoded state $\mathinner {|{\Psi }\rangle }_L$. When an error happens, one or more stabiliser measurements may yield −1. As there are four stabilisers whose measurement may take either +1 or −1 values, there are in total 15 syndrome measurement results with at least one outcome being −1. If we consider the ways in which a single Pauli error can afflict one of the five qubits, we note that there are 15 possibilities (three error types and five locations), with each mapping to a specific one of the 15 syndromes. When a two-qubit error happens, we again find that at least one of the stabiliser measurements takes −1. This heralds the fact that some error has occurred. However, since different double-qubit errors may have the same syndrome, we can only detect double-qubit errors without the capability of identifying or correcting them. Nevertheless, this latter property can be useful in some situations, such as state preparation, where we can simply discard a faulty realisation and restart.

**Figure 1.**
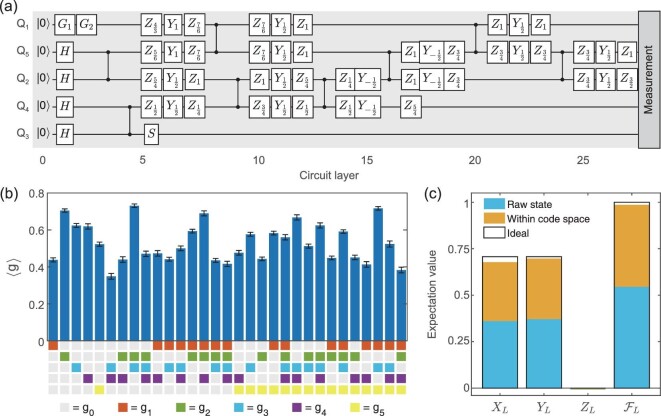
(a) Encoding quantum circuit of the five-qubit code. Here, the qubit labels Q_1_ ∼ Q_5_ are arranged to correspond with Equation (1); *G*_1_ and *G*_2_ are single-qubit gates to prepare the input state $a\mathinner {|{0}\rangle }+b\mathinner {|{1}\rangle }$ for encoding; *Y*_α_ and *Z*_α_ are the rotation gates around the *Y* and *Z* axes for an angle απ, respectively. In total, there are 27 layers of gate operations, including 54 single-qubit gates and eight nearest-neighbour controlled-phase gates. The single-qubit gates on different qubits can be applied in parallel, while the two-qubit gates can only be applied individually owing to the *Z* crosstalk. (b) Expectation values of 31 stabilisers for the encoded logical state $\mathinner {|{T}\rangle }_L$. Error bars representing a 95% confidence interval are estimated via bootstrapping. (c) Expectation values of logical Pauli operators and state fidelity of the encoded magic state.

Without using ancillary qubits, the original circuit for encoding the logical state $\mathinner {|{\Psi }\rangle }_L$ requires a number of two-qubit gates that are non-local with respect to a linear architecture [6,7]. To tailor the circuit for superconducting systems that only involve nearest-neighbour controlled-phase gates, we recompile the encoding circuit to have the minimal possible number (eight) of nearest-neighbour control-phase gates as shown in Fig. 1(a). We provide the details of circuit compilation in the online supplementary material.

## EXPERIMENTAL SETUP

The device for the implementation of the five-qubit error-correcting code is a 12-qubit superconducting quantum processor [28]. Among these 12 qubits, we chose five adjacent qubits to perform the experiment. The qubits are capacitively coupled to their nearest neighbours. The capacitively coupled *XY* control lines enable the application of single-qubit rotation gates by applying microwave pulses, and the inductively coupled *Z* control lines enable the double-qubit controlled-phase gates by adiabatically tuning the two-qubit state $\mathinner {|{11}\rangle }$ close to the avoid level crossing of $\mathinner {|{11}\rangle }$ and $\mathinner {|{02}\rangle }$ [28]. After careful calibrations and gate optimisations, we have the average gate fidelities as high as 0.9993 for single-qubit gates and 0.986 for two-qubit gates. With the implementation of only single-qubit rotation gates and double-qubit controlled-phase gates, we realised the circuit for encoding and decoding of the logical state. More details about the experimental setup are given in the online supplementary material.

## RESULTS

On a superconducting quantum processor [28], we experimentally realised the logical states $\mathinner {|{0}\rangle }_L$, $\mathinner {|{1}\rangle }_L$, $\mathinner {|{\pm }\rangle }_L$ and $\mathinner {|{\pm i}\rangle }_L$ that are eigenstates of the logical Pauli operators *X*_*L*_, *Y*_*L*_, *Z*_*L*_ and the magic state $\mathinner {|{T}\rangle }_L= (\mathinner {|{0}\rangle }_L+e^{i\pi /4}\mathinner {|{1}\rangle }_L)/\sqrt{2}$ that cannot be realised by applying Clifford operations on any eigenstate of the logical Pauli operators. The expectation values of the stabiliser operators of $\mathinner {|{T}\rangle }_L$ are shown in Fig. 1(b). The fidelity between the experimentally prepared state and the ideal state $\mathinner {|{\Psi }\rangle }_L\mathinner {\langle {\Psi }|}_L$ is determined by the measurement of the 32 stabiliser operators in $\prod _{i=1}^5(g_0+g_i)$. We omit the *g*_0_ one as it is constantly 1. In this way, we obtained the state fidelity as the average of the 32 stabilisers by picking up corresponding measurement results among the state tomography results. Finally, the state fidelity of $\mathinner {|{T}\rangle }_L$ reaches 54.5(4)%. The fidelities of other prepared states are shown in the online supplementary material, with an average fidelity of $57.1(3)\%$. The main error in preparing the encoded state is from decoherence, especially the relatively short dephasing time. In a numerical simulation of the experiment with decoherence (see the online supplementary material for details), the state fidelity of $\mathinner {|{T}\rangle }_L$ is $58.9\%$. After numerically increasing the dephasing time to be the same as the energy relaxation time, the state fidelity can be increased to $92.1\%$, indicating a potential direction for future improvements.

The quality of the prepared logical states can also be divided into its overlap with the logical code space and its agreement with the target logical state after projecting it into the code space. Given the logical Pauli operators *X*_*L*_, *Y*_*L*_, *Z*_*L*_ and $I_L=\mathinner {|{0}\rangle }_L\mathinner {\langle {0}|}_L+\mathinner {|{1}\rangle }_L\mathinner {\langle {1}|}_L$, the normalised density matrix ρ_*L*_ is defined by projecting the experimentally prepared state ρ_*q*_ into the code space

(2) \begin{equation*} \rho _L=\frac{I+\bar{P}_XX_L+\bar{P}_YY_L+\bar{P}_ZZ_L}{2}, \end{equation*}

with normalised probability $\bar{P}_j = P_j/P_I$ and *P*_*j*_ = Tr(ρ_*q*_σ_*L*_) for all σ = *I*, *X*, *Y*, *Z*, where ρ_*q*_ is the density matrix of the five-qubit state. We define the fidelity within the code space by $\mathcal {F}_L=\mathinner {\langle {\Psi }|}_L\rho _L\mathinner {|{\Psi }\rangle }_L$, as shown in Fig. 1(c), with the average value being as high as $98.6(1)\%$. Since projecting to the code space is equivalent to post-selecting all +1 stabiliser measurements, our result also indicates the possibility of high-fidelity logical state preparation with future non-destructive stabiliser measurements. This relies on whether we can achieve accurate non-destructive stabiliser measurements, especially whether errors from the ancillary qubits and additional gates are sufficiently low.

**Figure 2.**
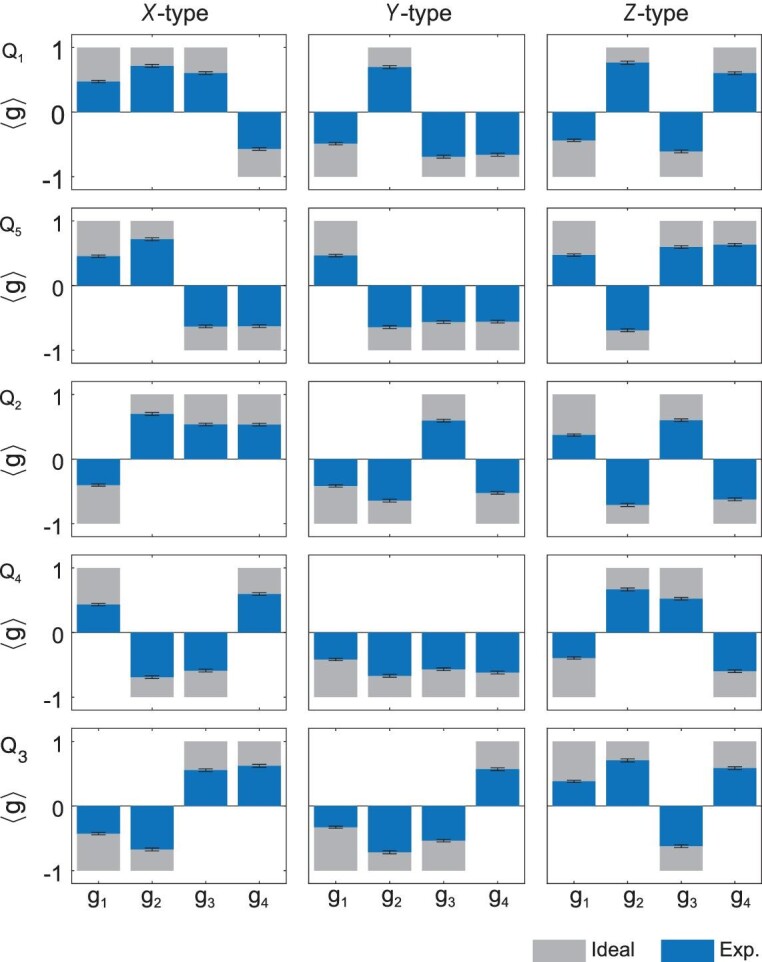
Destructive syndrome detection on the logic magic state $\mathinner {|{T}\rangle }_L$. A single-qubit *X*-, *Z*- or *Y*-type error, which corresponds to a bit-flip, phase-flip or combined error, respectively, is applied to one of the five qubits Q_1_ to Q_5_. We destructively measure the four stabilisers and find consistent syndrome correlations that identify the quantum error.

Given the realisation of the logical state, one can proceed to verification of the error correction/detection ability of the five-qubit code. Acting on the logical encoded state $\mathinner {|{T}\rangle }_L$, we systematically introduce every type of single-qubit error by artificially applying the corresponding single-qubit gate on one of the five qubits. Then, by measuring the four stabilisers *g*_1_, *g*_2_, *g*_3_ and *g*_4_, we aim to verify that each error would be properly identified. As shown in Fig. 2(a), for each case, we do indeed find the corresponding syndrome pattern that identifies the location of the single-qubit error. Suppose that the expectation value of *i*th stabiliser is *p*_*i*_; then the probability that the syndrome measurement works is $\prod $_*i*_(|*p*_*i*_| + 1)/2, which is 0.413 on average in experiment. We also apply double-qubit errors on $\mathinner {|{T}\rangle }_L$ and find the same syndrome correlation that can always detect the existence of errors (see the online supplementary material for details). Notably, the (single-qubit or double-qubit) error-afflicted states have probabilities projecting onto the code space (around $3.3\%$), verifying the power of the error-correcting code.

**Figure 3.**
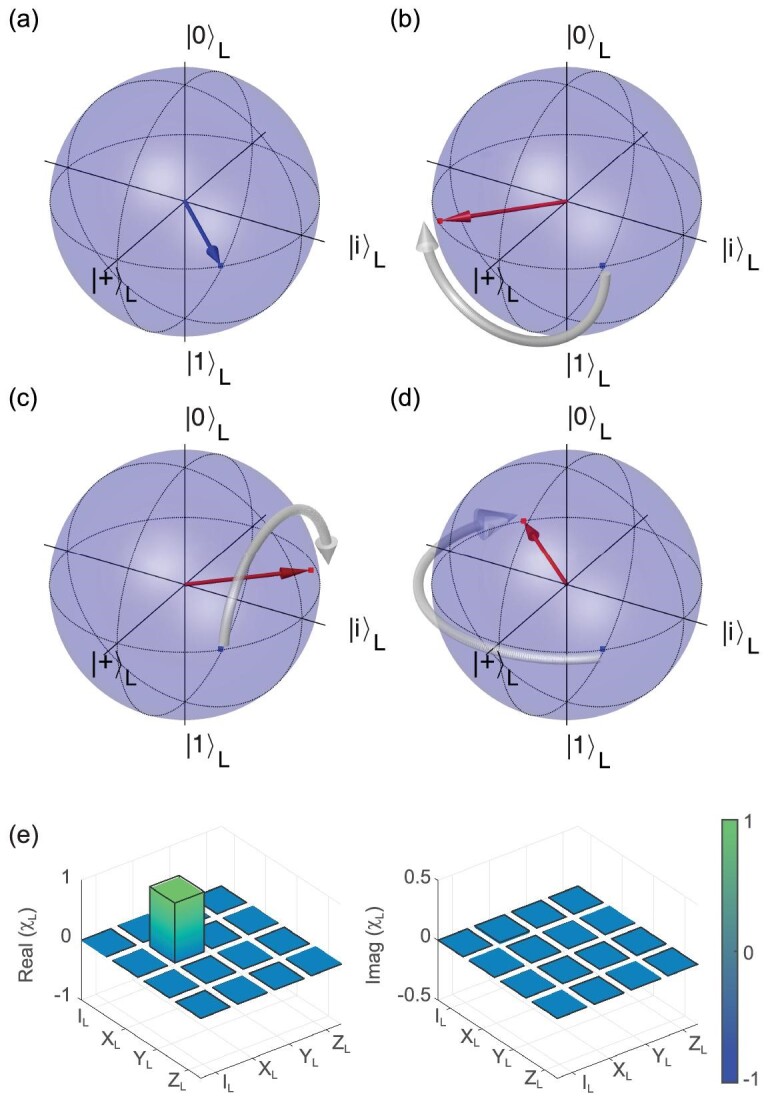
Logical operation within the code space. (a) Encoded logical state $\mathinner {|{T}\rangle }_L$ illustrated on the logical Bloch sphere. (b)–(d) Single logical-qubit operation *X*_*L*_, *Y*_*L*_ and *Z*_*L*_ applied on $\mathinner {|{T}\rangle }_L$. The blue squares and vector are the initial states. The red circles and vectors are the final states. The states are projected into the code space. The fidelities of the state after gate operation are 98.6(1)%, 98.0(1)% and 98.7(1)% for (b), (c) and (d), respectively. The white arrow illustrates the dynamics under the gate operation. (e) The χ_*L*_ matrix of the logical *X*_*L*_ operation determined via quantum process tomography in the code space. The gate fidelity of logical *X*_*L*_ operation is determined to be 97.2(2)%. The black-outlined hollow bars correspond to the ideal *X* gate. We refer the reader to the online supplementary material for the definition of the χ_*L*_ matrix and details.

In a functioning fault-tolerant quantum computer, operations on logical qubits are realised through a series of operations on the component physical qubits. We implement and verify three such transversal logical operations. Starting from the magic state $\mathinner {|{T}\rangle }_L$ presented in Fig. 3(a), we demonstrate the single logical-qubit operations *X*_*L*_, *Y*_*L*_ and *Z*_*L*_ and plot the rotated states within the code space, as shown in Fig. 3(b), (c) and (d), respectively. To characterise these logical operations, we performed the quantum process tomography within the code space as shown in Fig. 3(e), which reflects how well logical operations manipulate logical states. We determine gate fidelities of the logical *X*_*L*_, *Y*_*L*_ and *Z*_*L*_ operations to be $97.2(2)\%$, $97.8(2)\%$ and $97.3(2)\%$, respectively.

**Figure 4.**
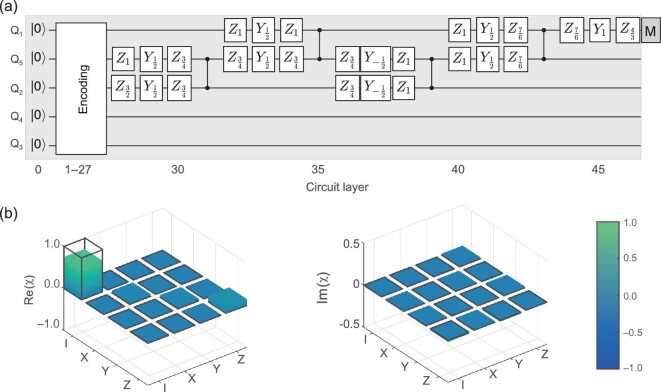
Decoding of the five-qubit code. (a) Decoding quantum circuit. After the logical state prepared with the encoding circuit shown in Fig. 1(b), we apply the decoding circuit to map the state back to a single-qubit state. The decoding circuit is essentially a reverse encoding circuit, except the gates applied on Q_3_ and Q_4_ are omitted because they do not affect the final decoded qubit. (b) The χ_*L*_ matrix of the encoding and decoding circuits. The color bars are the experimental χ_*L*_ matrix and the black-outlined hollow bars correspond to the identical process. The process fidelity reaches 74.5(6)%.

Finally, after encoding the single-qubit input state into the logical state, we apply the decoding circuit, see Fig. 4(a), to map it back to the input state. With input states $\mathinner {|{0}\rangle }$, $\mathinner {|{1}\rangle }$, $\mathinner {|{+}\rangle }$, and $\mathinner {|{+i}\rangle }$, we determine the state fidelity after decoding as 87.4(5)%, 91.6(4)%, 76.7(6)%, and 77.1(6)%, respectively. The relatively lower fidelities for $\mathinner {|{+}\rangle }$ and $\mathinner {|{+i}\rangle }$ states are also caused by the short dephasing time. After quantum process tomography from the four output states, the process fidelity is determined as $74.5(6)\%$, as shown in Fig. 4(b). The decoding circuit only applies operations on three qubits, highlighting the ability of quantum secret sharing with the five-qubit code [29]. This simplification is due to a consequence of locality: no observable on Q_1_ can be affected by the omitted independent gate operations of the other qubits.

## DISCUSSION

An essential milestone on the road to fault-tolerant quantum computing is the achievement of error-corrected logical qubits that genuinely benefit from error correction, outperforming simple physical qubits. There are three steps for achieving this goal: (1) realising encoded logical qubits in a code capable of detecting and correcting errors, (2) realising operations on encoded qubits and error-correction cycles and (3) adding more ancillary qubits and improving the operation fidelity to achieve fault tolerance. Our experiment completes step (1) by realising the basic ingredients of the full functional five-qubit error-correcting code. Our work partially achieves step (2) as we indeed perform logical operations and verify error detection; however, because we are only able to evaluate stabilisers destructively, we cannot perform full error correction. Directions for future works include the realisation of non-destructive error detection [25,26,30] and error correction, and the implementation of logical operations on multiple logical qubits for the five-qubit code. Our work also has applications in error mitigation for near-term quantum computing [31].

## DATA AVAILABILITY

All data analysed to evaluate the conclusions are available from the authors upon reasonable request.

## Supplementary Material

nwab011_Supplemental_File
