# Supplementary material for: Experimental exploration of five-qubit quantum error-correcting code with superconducting qubits
Source: Natl Sci Rev. 2021 Jan 21;9(1):nwab011. doi: 10.1093/nsr/nwab011 (PMC8776549; doi:10.1093/nsr/nwab011)
Supplement: nwab011_Supplemental_File [file nwab011_supplemental_file.pdf]

# Supplementary Data for "Experimental exploration of five-qubit quantum error correcting code with superconducting qubits"

## CONTENTS


I. Theory 1
 A. Code description 1
 B. Fidelity evaluation 2
 C. Error detection and correction 3
 D. Encoding circuit 3

II. Experiment 5
 A. Device 5
 B. Implementation of quantum circuits 5
 C. Logical state preparation 6
 D. Two-qubit error detection 7
 E. Logical gate operations 8
 F. Simulation of quantum circuits with decoherence 8

References 11


## I. THEORY

### A. Code description

The five-qubit quantum error correcting code (QECC) maps an input state $|\phi\rangle = a|0\rangle + b|1\rangle$ to a five-qubit state, $|\Psi\rangle_L = a|0\rangle_L + b|1\rangle_L$, where the logical $|0\rangle_L$ and $|1\rangle_L$ states are defined by

$$\begin{aligned}|0\rangle_L =&\frac{1}{4}[|00000\rangle + |10010\rangle + |01001\rangle + |10100\rangle\\&+|01010\rangle - |11011\rangle - |00110\rangle - |11000\rangle\\&-|11101\rangle - |00011\rangle - |11110\rangle - |01111\rangle\\&-|10001\rangle - |01100\rangle - |10111\rangle + |00101\rangle],\\|1\rangle_L =&\frac{1}{4}[|11111\rangle + |01101\rangle + |10110\rangle + |01011\rangle\\&+|10101\rangle - |00100\rangle - |11001\rangle - |00111\rangle\\&-|00010\rangle - |11100\rangle - |00001\rangle - |10000\rangle\\&-|01110\rangle - |10011\rangle - |01000\rangle + |11010\rangle].\end{aligned} \tag{S1}$$

The five-qubit code is a stabilizer code [1], which is defined by a set of independent operators from the Pauli group that have eigenvalues $\pm 1$. The stabilizer operators of the five-qubit code together with its logical Pauli operators are listed in Table S1.

The logical state space is defined by states $|\Psi\rangle_L = a|0\rangle_L + b|1\rangle_L$ which are simultaneously stabilized by the four stabilizers with $g_i|\Psi\rangle_L = +|\Psi\rangle_L$, $\forall i = 1,2,3,4$. Logical Pauli operators can be transversally realised by applying the corresponding operation separately on each physical qubit. The logical states $|0\rangle_L$ and $|1\rangle_L$ are eigenstates of the logical $Z_L$ operator. General logical operators, such as the $T_L = e^{-iZ_L\pi/8}$ gate, the Hadamard gate $H_L$, may not be transversally realised.

TABLE S1. The stabilizer generators of the five-qubit code and the logical $Z_L$, $X_L$, and $Y_L$ operations. Here $I_i$, $X_i$, $Y_i$, $Z_i$ are the Pauli matrices acting on the $i^{\text{th}}$ qubit.

| Name | Operator |
|---|---|
| $g_1$ | $X_1Z_2Z_3X_4I_5$ |
| $g_2$ | $I_1X_2Z_3Z_4X_5$ |
| $g_3$ | $X_1I_2X_3Z_4Z_5$ |
| $g_4$ | $Z_1X_2I_3X_4Z_5$ |
| $\overline{X}$ | $X_1X_2X_3X_4X_5$ |
| $\overline{Z}$ | $Z_1Z_2Z_3Z_4Z_5$ |
| $\overline{Y}$ | $Y_1Y_2Y_3Y_4Y_5$ |

## B. Fidelity evaluation

The logical state $|\Psi\rangle_L = a|0\rangle_L + b|1\rangle_L$ can be uniquely determined by the four stabilizers defined in Table S1 together with the fifth stabilizer

$$\begin{aligned} g_5 &= |\Psi\rangle\langle\Psi| - |\Psi^\perp\rangle\langle\Psi^\perp| \\ &= (a|0_L\rangle + b|1_L\rangle)(a^\star\langle 0_L| + b^\star\langle 1_L|) - (b^\star|0_L\rangle - a^\star|1_L\rangle)(b\langle 0_L| - a\langle 1_L|) \\ &= (aa^\star - bb^\star)(|0_L\rangle\langle 0_L| - |1\rangle\langle 1_L|) + 2a^\star b|1_L\rangle\langle 0_L| + 2b^\star a|0_L\rangle\langle 1_L| \\ &= (aa^\star - bb^\star)Z_L + (a^\star b + b^\star a)X_L - i(a^\star b - b^\star a)Y_L. \end{aligned} \tag{S2}$$

with $|\Psi^\perp\rangle = b^\star|0_L\rangle - a^\star|1_L\rangle$. That is, any logical state $|\Psi\rangle_L$ can be decomposed as

$$|\Psi\rangle_L\langle\Psi|_L = \frac{1}{2^5}\prod_{i=1}^{5}(g_0 + g_i), \tag{S3}$$

where $g_0 = I_1I_2I_3I_4I_5$. Therefore the fidelity between the experimentally prepared state $\rho_q$ and the ideal target state $|\Psi\rangle_L\langle\Psi|_L$ can be also determined by the measurement of the 32 stabilizer operators $\prod_{i=1}^{5}(g_0 + g_i)$. That is,

$$\mathcal{F} = \langle\Psi|_L\rho_q|\Psi\rangle_L = \sum_j \mathrm{tr}[\rho_q g_j], \tag{S4}$$

where $g_j$ is one of the 32 terms by expanding $\prod_{i=1}^{5}(g_0 + g_i)$.

The fidelity of the prepared logical states can also be divided into its overlap with the logical code space and its agreement with the target logical state after projecting it into the code space. Given the logical Pauli operators,

$$\begin{aligned} X_L &= |+\rangle_L\langle +|_L - |-\rangle_L\langle -|_L, \\ Y_L &= |+i\rangle_L\langle +i|_L - |-i\rangle_L\langle -i|_L, \\ Z_L &= |0\rangle_L\langle 0|_L - |1\rangle_L\langle 1|_L, \\ I_L &= |0\rangle_L\langle 0|_L + |1\rangle_L\langle 1|_L, \end{aligned} \tag{S5}$$

with $|\pm\rangle_L = (|0\rangle_L \pm |1\rangle_L)/\sqrt{2}$ and $|\pm i\rangle_L = (|0\rangle_L \pm i|1\rangle_L)/\sqrt{2}$, the normalised density matrix $\rho_L$ is defined by projecting $\rho_q$ into the code space

$$\rho_L = \frac{I + \bar{P}_X X_L + \bar{P}_Y Y_L + \bar{P}_Z Z_L}{2}, \tag{S6}$$

with normalised probability $\bar{P}_j = P_j/P_I$ and $P_j = \mathrm{Tr}(\rho_q j_L)$, for all $j = I$, $X$, $Y$, $Z$. After projecting $\rho_q$ into the code space, we define the fidelity within the code space by

$$\mathcal{F}_L = \langle\Psi|_L\rho_L|\Psi\rangle_L. \tag{S7}$$

### C. Error detection and correction

The five-qubit code has a distance three, and therefore any single-qubit or two-qubit errors will be detected by a stabilizer change, and single-qubit errors can be successfully corrected (while if the correction protocol were applied to a two-qubit error state, a logical error would result). When there is no error, the stabilizers are all 1 for the encoded state $|\Psi\rangle_L$. When error happens, the stabilizers may have negative values. As there are four stabilizers either taking $\pm 1$ values, there are in total 15 syndrome measurement results with at least one stabilizer value being negative. For each one of the 15 cases with one single qubit error happens on one of the five qubits, it corresponds to a unique syndrome. When a two-qubit error happens, one of the stabilizers must take a negative value, indicating the existence of errors. As different two-qubit errors may have the same syndrome (degeneracy), we can only detect two-qubit errors without the capability of correcting them.

### D. Encoding circuit

In our experiment, we also consider the case where we relabel the code by mapping the original labels of the qubits as follows,

$$1' = 1,\ 2' = 5,\ 3' = 2,\ 4' = 4,\ 5' = 3. \tag{S8}$$

The four stabilizers of the relabeled code are summarized in Table S2.

TABLE S2. The stabilizer generators of the relabelled five-qubit QECC code.

| Name | Operator |
|---|---|
| $g_1$ | $X_1Z_2Z_3X_4I_5 = X_{1'}I_{2'}Z_{3'}X_{4'}Z_{5'}$ |
| $g_2$ | $I_1X_2Z_3Z_4X_5 = I_{1'}X_{2'}X_{3'}Z_{4'}Z_{5'}$ |
| $g_3$ | $X_1I_2X_3Z_4Z_5 = X_{1'}Z_{2'}I_{3'}Z_{4'}X_{5'}$ |
| $g_4$ | $Z_1X_2I_3X_4Z_5 = Z_{1'}Z_{2'}X_{3'}X_{4'}I_{5'}$ |

The encoding circuit of the conventional five-error correction code is shown in Fig. S1. We manually searched this circuit so that minimal number of Controlled-Not (CNOT) gates is used. Here the gates that are used in the circuit are defined as

$$H = \frac{1}{\sqrt{2}}\begin{bmatrix}1 & 1\\ 1 & -1\end{bmatrix}, S = \begin{bmatrix}1 & 0\\ 0 & i\end{bmatrix}, X = \begin{bmatrix}0 & 1\\ 1 & 0\end{bmatrix}, Z = \begin{bmatrix}1 & 0\\ 0 & -1\end{bmatrix}. \tag{S9}$$

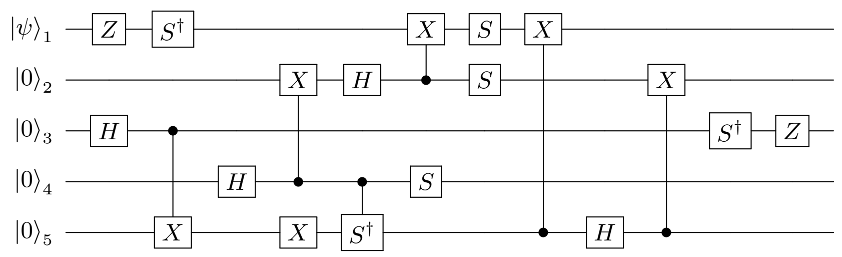


FIG. S1. Encoding circuit for the five-qubit error correction code. Qubit 1 is the unknown to-be encoded state $a|0\rangle + b|1\rangle$ and qubit 2 to 5 are the ancillae $|0\rangle$.

In our experiment, we relabel the qubits from $1, 2, 3, 4, 5$ to $1', 3', 5', 4', 2'$ in order to make the nonlocal gates to be nearest-neighbour gates. By reordering the qubits as $1', 2', 3', 4', 5'$, we obtain the encoding circuit that only involves 6 nearest-neighbour gates and 2 swap gates, shown in Fig. S2. Each swap gate can be realized with 3 CNOT gates. The total number of nearest-neighbour CNOT gates are 12. It is worth noting that relabelling or reordering the ancillary qubits do not affect the code. The code is equivalent simply with stabilizers and all other measurement reordered.

Furthermore, we show that the number of nearest-neighbour CNOT gates can be reduced to 8 by numerically optimising the circuit. This is achieved by focusing on two clusters of gates in the two dashed boxes. As shown in

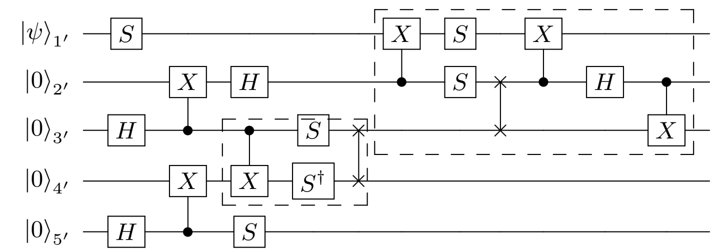

FIG. S2. Encoding circuit for the relabelled five-qubit error correction code.

Fig. S3, we recompile these two clusters of gates by replacing the swap gate with the Control-$Z$ (CZ) gate and in the meanwhile inserting more parameterized single qubit gates. For each single qubit gate, we decompose it as

$$U_i = R_z(\alpha_i)R_y(\beta_i)R_z(\gamma_i), \tag{S10}$$

with $R_z(\alpha) = e^{-i\alpha\sigma_z/2}$ and $R_y(\alpha) = e^{-i\alpha\sigma_y/2}$. We also add a parameter to the unitary to represent its global phase. Suppose the target unitary is $U$ and the parameterised compiled circuit is $U(\vec{\theta})$, with $\vec{\theta}$ denoting all the parameters. Then we need to minimise the distance between $U$ and $U(\vec{\theta})$

$$\min_{\vec{\theta}} \|U(\vec{\theta}) - U\|, \tag{S11}$$

where $\|U\| = \sum_{i,j} |U_{i,j}|^2$. We numerically optimise the distance over all the parameters and we find that the circuits of the two dashed boxes can be simplified as desired with four CZ or CNOT gates reduced.

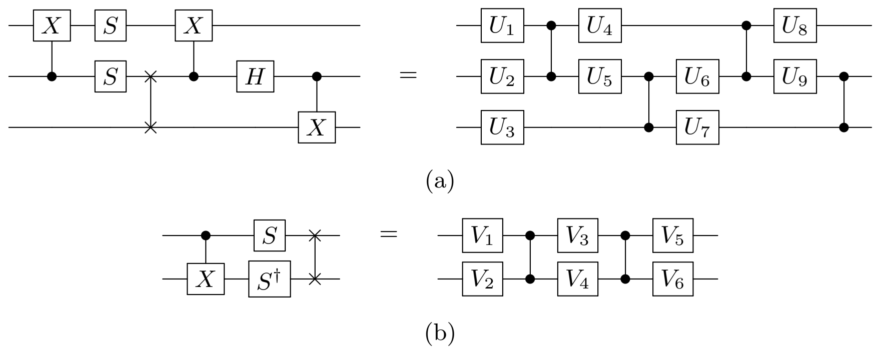

FIG. S3. Quantum circuit compilation. We recompile the two small circuits by fixing its structure with several parameterized single qubit gates. We replace the swap gate with the CZ gate and add general single qubit gates between every two CZ gates. Each single qubit gate has three parameters and we also add a global phase to the whole circuit. Circuit (a) has 28 parameters and Circuit (b) has 19 parameters. We perform the numerical optimization with the MATLAB *fminsearch* function, which realized the simulated annealing algorithm. As the parameter space is quite large, we run the algorithm with random initial parameters and find a local minimum at each time. We end the search until the distance is minimized that is below a certain threshold, say $10^{-3}$. In practice, we only need to try tens of random initial parameters to find the global minimum. In general, the found rotation angles are not the exact factional number as shown in Fig. S4 owing to numerical accuracy. We manually replace the rotation angles with the closest fractional numbers and verify the compiled circuit by obtaining negligible distance, say $10^{-10}$.

After compiling the circuit and combining single qubit gates, we obtain our final encoding circuit as shown in Fig. S4.

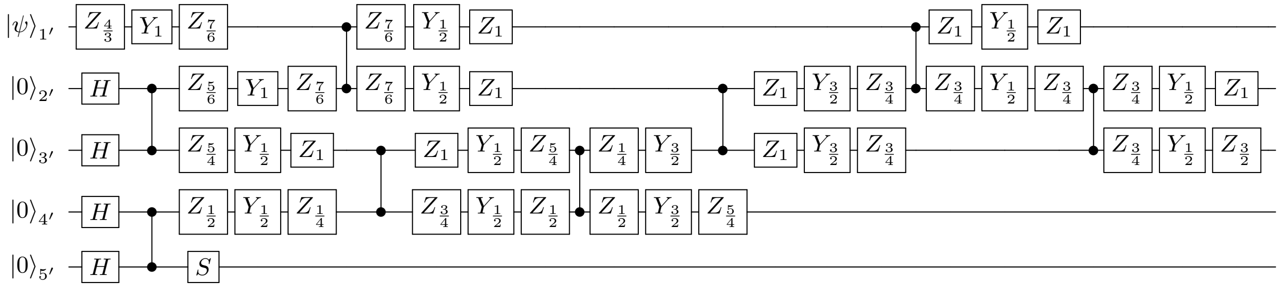

FIG. S4. Encoding circuit for the five qubit error correction code. Here $X_\alpha = e^{-i\alpha\sigma_x/2}$, $Y_\alpha = e^{-i\alpha\sigma_y/2}$, and $Z_\alpha = e^{-i\alpha\sigma_z/2}$ with Pauli matrices $\sigma_x$, $\sigma_y$, and $\sigma_z$.

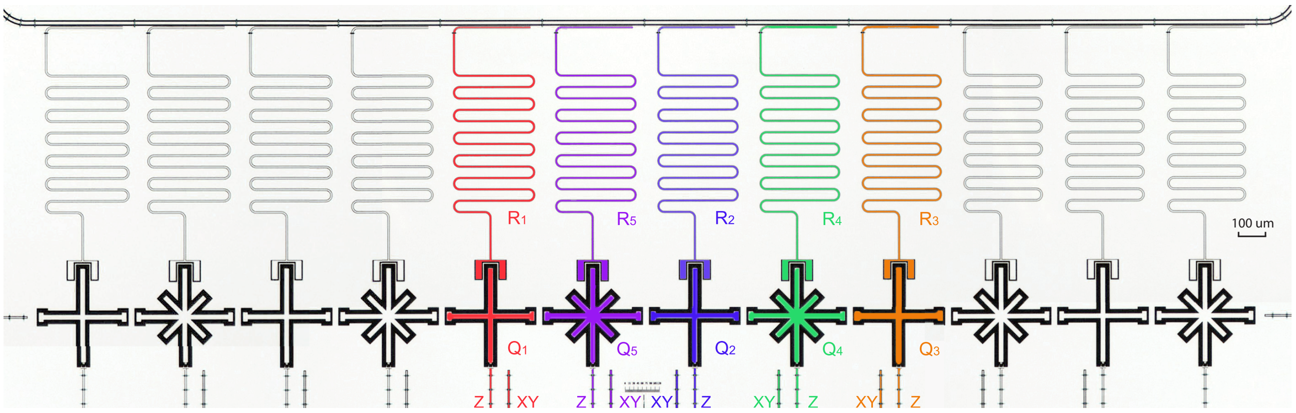


FIG. S5. False-color optical image of the superconducting quantum processor. There are in total 12 qubits, from which we choose five adjacent qubits labeled with $Q_1$ to $Q_5$ to perform the experiment. Each qubit couples to a corresponding resonator for state readout.

## II. EXPERIMENT

### A. Device

The device we used is a superconducting quantum processor. As illustrated in Fig. S5, there are 12 transmon qubits of the Xmon variety [2–4] arranged in a 1D chain [5]. All qubits are frequency-tunable by their corresponding Z control lines. Each qubit couples to its nearest-neighbour qubits via fixed capacitors. The nearest-neighbour coupling strength is about 12 MHz. For each qubit, individual XY and Z control lines enable the ability to fully control the qubit state. Each qubit couples to a $\lambda/4$ resonator for state readout. All twelve resonators couple to a common transmission line. Among these qubits, we choose five high-quality adjacent qubits, labelled from $Q_1$ to $Q_5$ in Fig. S5, to perform the experiment. The performances of the qubits are listed in Table. S3. The relaxation time $T_1$ ranges from 27.5 $\mu$s to 48.6 $\mu$s. The dephasing time $T_2^*$ ranges from 2.7 $\mu$s to 5.6 $\mu$s. To reduce the $ZZ$ coupling between the neighboring qubits, the idle frequencies of the qubits alternate in a zigzag pattern. The minimum frequency difference between neighboring qubits is 740 MHz. A schematic diagram of the experimental wiring setup is shown in Fig. S6.

### B. Implementation of quantum circuits

The single-qubit rotation gates around $X$- or $Y$-axis are realized by applying Gaussian-enveloped microwave pulses through the $XY$ control lines. A derivative reduction by adiabatic gate (DRAG) [6] protocol is used to reduce the phase error and state leakage to the second excited state in the application of single qubit gates. Note that for the realization of the single-qubit rotation gates around the $Z$-axis, i.e., $R_z(\theta)$, we shift the phase of the reference for a certain angle $\theta$ instead of applying a physical detuning pulse. The controlled-phase gates used in our experiment is the fast adiabatic CZ gates [3, 7], realized by tuning the two qubit $|11\rangle$ state close to the avoid-crossing of $|11\rangle$ and $|02\rangle$

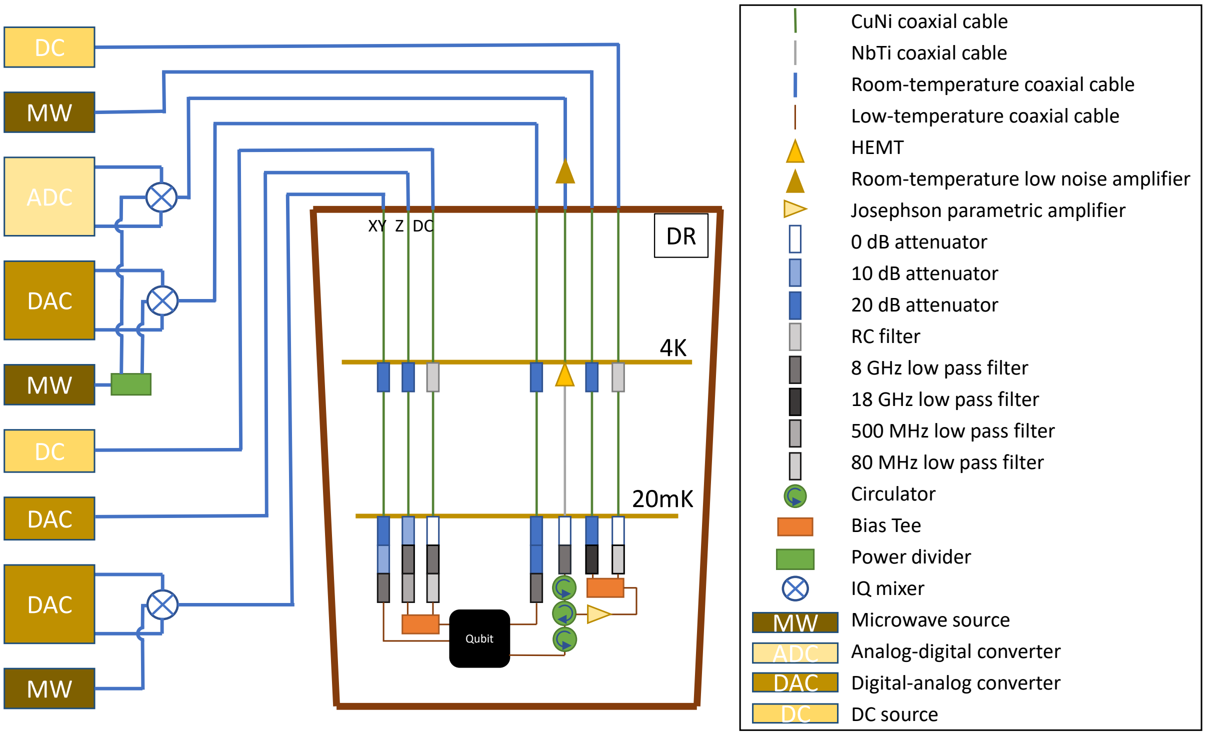


FIG. S6. Schematic diagram of the experimental wiring setup. For each qubit, there are individual XY, Z and DC control lines. The Z and DC lines are combined via bias tees before connected to the quantum processor. We use microwave (MW) sources, IQ mixers and digital-analog converters (DACs) to generate XY control signals. The Z control signals are realized by the DACs. The qubits are flux-biased at their idle points by the DC sources. The state of different qubit can be read out simultaneously using frequency multiplexing. A Josephson parametric amplifier is connected to the second cryogenic circulator. A HEMT is used on the 4K plate for readout amplification. The signal is further amplified via a room-temperature amplifier and then demodulated and digitized by an analog-digital converter (ADC). The setups of attenuators and filters for different control lines are different to minimize the noise.

state following an adiabatic trajectory. For calibration of the two-qubit gates, the first step is to correct the z pulse cross talk and z pulse distortion [5, 8]. After that, we choose the operation point avoiding noticeable two level systems (TLSs). After the operation point is chosen, we use Nelder-Mead algorithm to optimize the waveform parameters, including the gate amplitude, length, and the parameters controlling the shape of the waveform. The cost function is chosen as the process fidelity estimated via quantum process tomography. We cannot use RB as the cost function as the dynamical phase for the qubits is not determined when optimizing the waveform parameters. At last, we measure the dynamical phases of all relevant qubits. The single- and double-qubit gate fidelities, determined via interleaved randomized benchmarking (RB), are listed in Table. S3, with the average fidelity obtained as 99.93% and 98.6%, respectively. The experimental waveform sequences are shown in Fig. S7. The evolution time for both encoding and decoding processes are about 810 ns. For each result measured in our experiment, we repeat the waveform sequences and state readout for 5,000 to 10,000 times.

### C. Logical state preparation

We prepare seven logical states, including the six eigenstates $|0\rangle_L$, $|1\rangle_L$, $|+\rangle_L = (|0\rangle_L + |1\rangle_L)/\sqrt{2}$, $|-\rangle = (|0\rangle_L - |1\rangle_L)/\sqrt{2}$, $|+i\rangle = (|0\rangle_L + i\,|1\rangle_L)/\sqrt{2}$, and $|-i\rangle = (|0\rangle_L - i\,|1\rangle_L)/\sqrt{2}$ of the Pauli matrices and the magic state $|T\rangle_L = (|0\rangle_L + e^{i\pi/4}\,|1\rangle_L)/\sqrt{2}$. The results are shown in Table S4, where the raw state fidelities are determined as the fidelity between the experimentally obtained density matrix and that of the ideal state. Focusing on states in the code space, the state fidelity can be enhanced from 55.3% to 98.6% on average. The probability of projecting the state to the code space is 56.2%, which is close to the raw state fidelity after multiplying the fidelity in the code space. The expectation values of logical Pauli operators and the state fidelity of the magic state $|T\rangle_L$ are shown in Fig. 1 (c) in main text. The results for the other six states are shown in Fig. S8.

| Qubit | $Q_1$ | $Q_5$ | $Q_2$ | $Q_4$ | $Q_3$ | AVG. |
|---|---|---|---|---|---|---|
| $\omega_{10}/2\pi$ (GHz) | 5.124 | 4.266 | 5.006 | 4.134 | 4.884 | - |
| $T_1$ ($\mu s$) | 27.5 | 34.0 | 33.0 | 36.8 | 48.6 | 36.0 |
| $T_2^*$ ($\mu s$) | 5.5 | 4.1 | 5.6 | 2.7 | 3.3 | 4.2 |
| $f_{00}$ | 0.982 | 0.932 | 0.931 | 0.934 | 0.963 | 0.945 |
| $f_{11}$ | 0.831 | 0.874 | 0.885 | 0.899 | 0.916 | 0.872 |
| X/2 gate errors per Clifford sequence for the reference | 0.0014 | 0.0017 | 0.0024 | 0.0018 | 0.0015 | |
| X/2 gate errors per Clifford sequence for the interleaved RB | 0.0020 | 0.0023 | 0.0031 | 0.0025 | 0.0022 | |
| X/2 gate fidelity | 0.9994 | 0.9994 | 0.9992 | 0.9993 | 0.9993 | 0.9993 |
| CZ gate errors per Clifford sequence for the reference | 0.037 | 0.035 | 0.038 | 0.026 | | |
| CZ gate errors per Clifford sequence for the interleaved RB | 0.056 | 0.045 | 0.053 | 0.038 | | |
| CZ gate fidelity | 0.980 | 0.990 | 0.984 | 0.988 | | 0.986 |

TABLE S3. Performance of qubits. $\omega_{10}$ is idle points of the qubits. $T_1$ and $T_2^*$ are the energy relaxation time and dephasing time, respectively. $f_{00}$ ($f_{11}$) is the probability of correctly readout of qubit state in $|0\rangle$ ($|1\rangle$) after successfully initialized in $|0\rangle$ ($|1\rangle$) state. X/2 gate fidelity and CZ gate fidelity are single- and two-qubit gate fidelities obtained via performing randomized benchmarking.

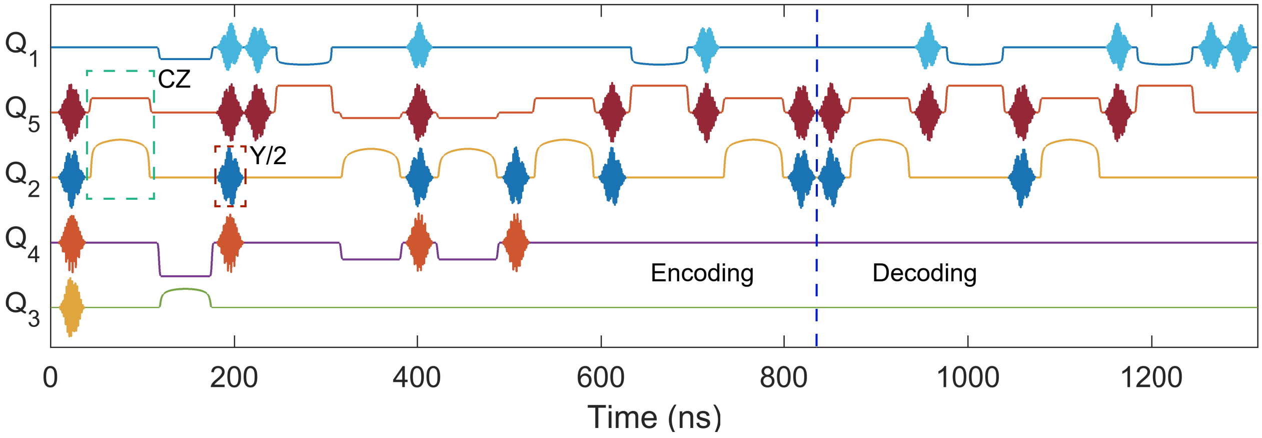


FIG. S7. Experimental waveform sequences for the implementation of encoding-decoding process. A typical CZ gate and a Y/2 gate are marked in the green and red dashed boxes, respectively. The blue dashed line marks the joint between encoding, idle and decoding processes.

## D. Two-qubit error detection

The result of single qubit error identification is shown in the main text. We found that the measured syndrome correlation can uniquely determine the artificially introduced single error. To check the two-qubit error detection with the five-qubit error correction code, we firstly prepared the logical encoded state $|T_L\rangle$. Then, by applying two single-qubit gates on two of the five qubits, we introduce artificial two-qubit errors. The single-qubit gates are chosen

TABLE S4. Fidelity of the prepared logical states. The state fidelity within code space is equivalent to the one of the state after post-selecting +1 stabilizer measurement outcomes. The uncertainties are estimated via bootstrapping.

| Logical state | $\|0\rangle_L$ | $\|1\rangle_L$ | $\|+\rangle_L$ | $\|-\rangle_L$ | $\|+i\rangle_L$ | $\|-i\rangle_L$ | $\|T\rangle_L$ | AVG. |
|---|---|---|---|---|---|---|---|---|
| State fidelity from state tomography | 0.567(3) | 0.533(3) | 0.527(3) | 0.581(3) | 0.594(3) | 0.547(3) | 0.524(4) | 0.553(3) |
| State fidelity from stabilizers | 0.586(3) | 0.551(3) | 0.541(3) | 0.598(3) | 0.612(3) | 0.564(3) | 0.545(4) | 0.571(3) |
| State fidelity within code space | 0.984(1) | 0.988(1) | 0.982(1) | 0.990(1) | 0.987(1) | 0.975(1) | 0.993(1) | 0.986(1) |
| Post-selection probability | 57.6% | 54.0% | 53.6% | 58.7% | 60.2% | 56.2% | 53.2% | 56.2% |

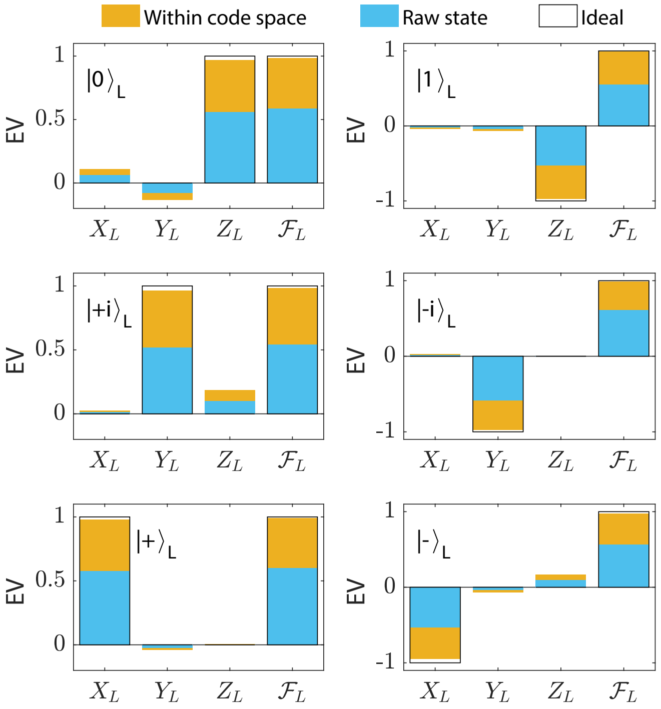


FIG. S8. Expectation values of logical Pauli operators and state fidelity of logical states. The six prepared states are $|0\rangle_L$, $|1\rangle_L$, $|+i\rangle_L$, $|-i\rangle_L$, $|+\rangle_L$, and $|-\rangle_L$, respectively. In each plot, logical Pauli expectation values and the state fidelity for the ideal state, raw experiment states, and states within the code space are shown in black-outlined hollow, blue, and brown bars, respectively.

from the $X$, $Z$ and $Y$ gates. We measured the four stabilizer operators $g_1$, $g_2$, $g_3$, and $g_4$, and realized the error detection of the two-qubit error. The exact syndrome indicates that the existence of two-qubit errors can be exactly detected. The results are shown in Fig. S9.

### E. Logical gate operations

The fidelity of logical gate operations are determined by performing quantum process tomography(QPT) [9, 10] of the corresponding operations. The $\chi$ matrix determined in QPT is defined as $\varepsilon(\rho) = \Sigma E_m \rho E_n^\dagger \chi_{mn}$, where $\varepsilon$ is the quantum operation, $\rho$ is the density matrix of the input state, and $E_m$'s are the operation bases, which in our case corresponds to $\{I, \sigma_x, -i\sigma_y, \sigma_z\}$. We prepare 4 input states of $Q_1$ in total, i.e. $|0\rangle$, $|1\rangle$, $|+\rangle$, and $|+i\rangle$, and measure the quantum state tomography (QST) of the output state of $Q_1$ with each input state. For each quantum state tomography components, we repeat the gate sequences and measurement for 5,000 times. After correcting the measurement error of QST with maximum-likelihood estimation, we reconstruct the 4×4 experimental $\chi$ matrix from the 4 density matrices. The process fidelity is determined as the trace overlap between the $\chi$ matrices of the ideal process and that obtained from QPT. The raw gate fidelities are determined to be 86.8%, 87.2%, and 86.1%, for $X_L$, $Y_L$, and $Z_L$, respectively. Instead, we can also consider gate fidelities only in the code space. For each state tomography, we extract the density matrix of the state within the code space. For $Y_L$ and $Z_L$, the gate fidelities in the code space are determined to be 97.8(2)% and 97.3(2)%, respectively. After the logical $X_L$, $Y_L$ , and $Z_L$ operations , the average probability of staying within the code space for logical states $|0\rangle_L$, $|1\rangle_L$, $|+\rangle_L$, and $|i\rangle_L$ is 0.576, 0.568, 0.541, and 0.566, respectively. The results are shown in Fig. S10.

### F. Simulation of quantum circuits with decoherence

We use operator-sum representations to simulate the evolution of the system with relaxation and dephasing[11]. After replacing ideal quantum gates with Kraus operators, the evolution of the system with quantum gate G applied

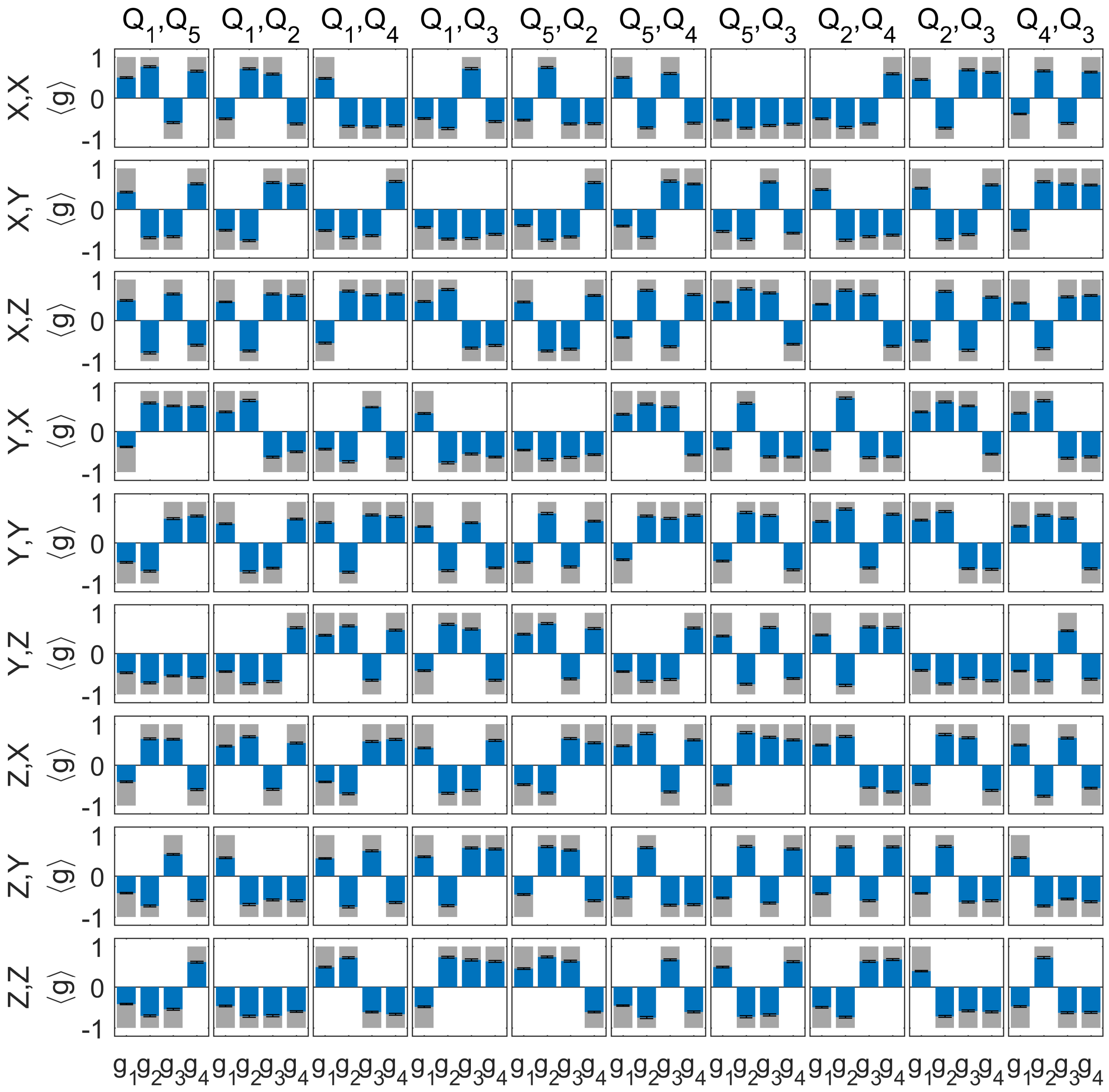


FIG. S9. Two-qubit error detection. Each row corresponds to a type of two-qubit error, and each column corresponds to a set of qubits on which coherent error applied. The exact syndrome patterns indicate the ability of detecting double-qubit errors.

to can be written as:

$$\varepsilon(\rho) = \sum_k E_k G \rho G^\dagger E_k^\dagger \tag{S12}$$

where G is the ideal quantum gate, $E_k$s are the operation elements representing decoherence, and $E_k G$ constitutes one of the Kraus operators.

The matrix forms are different for different kinds of decoherence. For relaxation, the matrix forms are

$$E_1 = \begin{bmatrix} 1 & 0 \\ 0 & \sqrt{1-\gamma} \end{bmatrix} \qquad E_2 = \begin{bmatrix} 0 & \sqrt{\gamma} \\ 0 & 0 \end{bmatrix} \tag{S13}$$

where $\gamma$ is the probability of losing a exciton within the gate time and thus corresponds to the ratio of the gate time

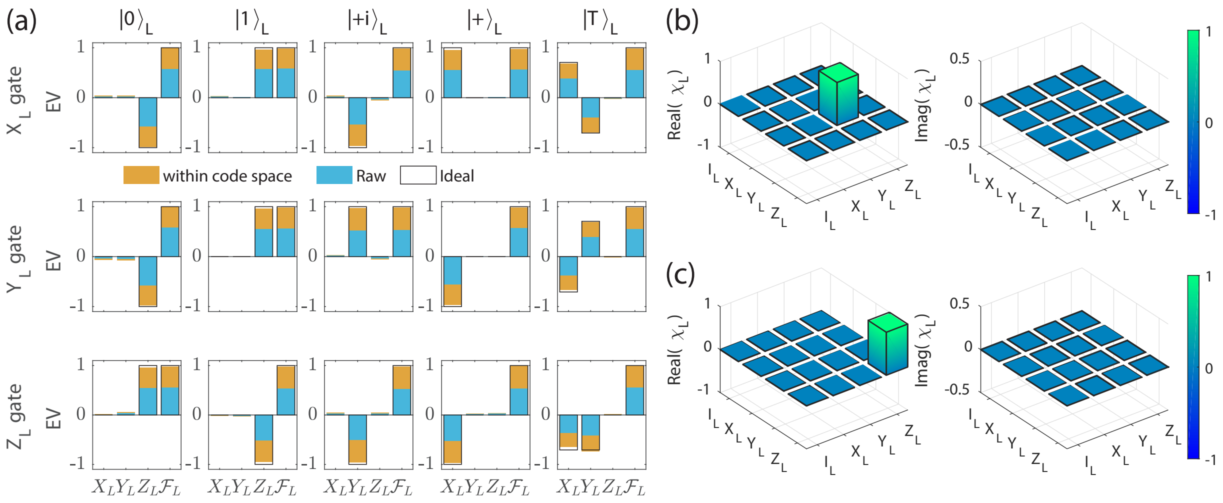


FIG. S10. (a) Expectation values of three logical Pauli operators and the state fidelity of the five initial logical states after the corresponding logical qubit operation, $X_L$, $Y_L$, and $Z_L$, respectively. The five logical states are $|0\rangle_L$, $|1\rangle_L$, $|+\rangle_L$, $|+i\rangle_L$, $|T\rangle_L$, respectively. For each gate operation, the state tomography with the first four initial states are used to obtain the $\chi_L$ matrix of the process. In each figure, logical qubit density matrix determined with post-selection, without post-selection, and that of the ideal state, are shown in brown, blue, and black outlined hollow bars, respectively. (b) and (c) Quantum process tomography of logical operations $Y_L$ and $Z_L$, respectively. The logical qubit density matrices are determined with post-selection. The process fidelities are 97.8(2)% and 97.3(2)%, respectively. The black-outlined hollow bars correspond to the ideal process.

TABLE S5. Numerical simulation results for encoding and decoding. For the rows labeled "Original decoherence", we use the decoherence information from Table. S3. For the rows labeled "Long $T_2 = T_1$", we numerically increase the $T_2$ to be the same as $T_1$.

| State fidelities of the encoded states | | | | | | | | |
|---|---|---|---|---|---|---|---|---|
| Logical state | $\|0\rangle_L$ | $\|1\rangle_L$ | $\|+\rangle_L$ | $\|-\rangle_L$ | $\|+i\rangle_L$ | $\|-i\rangle_L$ | $\|T\rangle_L$ | AVG. |
| Original decoherence | 0.607 | 0.607 | 0.589 | 0.589 | 0.589 | 0.589 | 0.589 | 0.594 |
| Long $T_2 = T_1$ | 0.924 | 0.924 | 0.921 | 0.921 | 0.921 | 0.921 | 0.921 | 0.922 |

| State and process fidelities of decoding | | | | | |
|---|---|---|---|---|---|
| Initial state | $\|0\rangle$ | $\|1\rangle$ | $\|+\rangle$ | $\|+i\rangle$ | Process fidelity |
| Original decoherence | 0.915 | 0.916 | 0.847 | 0.836 | 0.799 |
| Long $T_2 = T_1$ | 0.971 | 0.972 | 0.958 | 0.961 | 0.945 |

to the relaxation time $T_1$ ($\gamma = k\dfrac{t_{gate}}{T_1}$). In this work, we set $k$ equal to 1. For dephasing, the matrix forms are

$$E_3 = \begin{bmatrix} 1 & 0 \\ 0 & \sqrt{1-\gamma_\phi} \end{bmatrix} \qquad E_4 = \begin{bmatrix} 0 & 0 \\ 0 & \sqrt{\gamma_\phi} \end{bmatrix} \tag{S14}$$

where $\gamma_\phi$ is the probability that the exciton being scattered within the gate time and thus proportional to the ratio of the gate time to the dephasing time $T_\phi$ ($\gamma_\phi = k'\dfrac{t_{gate}}{T_\phi}$). We set the scale factor $k'$ to 2 considering no spin echoes applied in this work.

Taking both relaxation and dephasing into account, we combine these operation elements into a new element set $E_1' = E_1E_3$, $E_2' = E_1E_4$, $E_3' = E_2E_3$, $E_4' = E_2E_4$.

In this way, we can numerically simulate the evolution of designed quantum circuits with decoherence. As shown in Table. S5, the numerical simulation results for both encoding and decoding are close to the experimental results, indicating that the main error in our experiment is decoherence. Moreover, after numerically increasing the dephasing time to be the same as the energy relaxation time, the state fidelities of encoded states and process fidelity of decoding increase significantly.

We then further calculate the fidelity between the observed state and the numerically simulated state. For $|T_L\rangle$ state, the fidelity is 0.489. The fidelity is different from 1 might owe to the incomplete estimation of the noise

model in the experiment. In the current noise model, only the decoherence has been considered. The coherent error, especially the control errors in two-qubit gates are not taken into account, which may lead to errors in the final output state. By adding single-qubit phase to the numerically simulated state, the fidelity can be improved from 0.489 to 0.518. Moreover, as shown in Fig. S11, by partial tracing the qubits in the final state, we found that the single-qubit state fidelity is very high ($\sim$99%) and decay very fast with the increasing of the qubit number, indicating that the multi-qubit coherent error might be one of the main error sources.


[1] Gottesman D. Class of quantum error-correcting codes saturating the quantum hamming bound. *Phys Rev A* 1996; **54**: 1862–8.

[2] Barends R, Kelly J and Megrant A *et al.* Coherent josephson qubit suitable for scalable quantum integrated circuits. *Phys Rev Lett* 2013; **111**: 1–6.

[3] Barends R, Kelly J and Megrant A *et al.* Superconducting quantum circuits at the surface code threshold for fault tolerance. *Nature* 2014; **508**: 500–3.

[4] Kelly J, Barends R and Fowler AG *et al.* State preservation by repetitive error detection in a superconducting quantum circuit. *Nature* 2015; **519**: 66-9.

[5] Gong M, Chen MC and Zheng Y *et al.* Genuine 12-Qubit Entanglement on a Superconducting Quantum Processor. *Phys Rev Lett* 2019; **122**: 110501.

[6] Lucero E, Kelly J and Bialczak RC *et al.* Reduced phase error through optimized control of a superconducting qubit. *Phys Rev A* 2010; **82**: 1–7.

[7] Martinis JM and Geller MR. Fast adiabatic qubit gates using only $\sigma_z$ control. *Phys Rev A* 2014; **90**: 1–9.

[8] Yan Z, Zhang YR and Gong M *et al.* Strongly correlated quantum walks with a 12-qubit superconducting processor. *Science* 2019; **364**: 753–6.

[9] Chuang IL and Nielsen MA. Prescription for experimental determination of the dynamics of a quantum black box. *J Mod Opt* 1997; **44**: 2455–67.

[10] Poyatos JF, Cirac JI and Zoller P. Complete characterization of a quantum process: The two-bit quantum gate. *Phys Rev Lett* 1997; **78**: 390–3.

[11] Nielsen MA and Chuang IL. *Quantum Computation and Quantum Information: 10th Anniversary Edition.*, New York: Cambridge University Press, 2010.

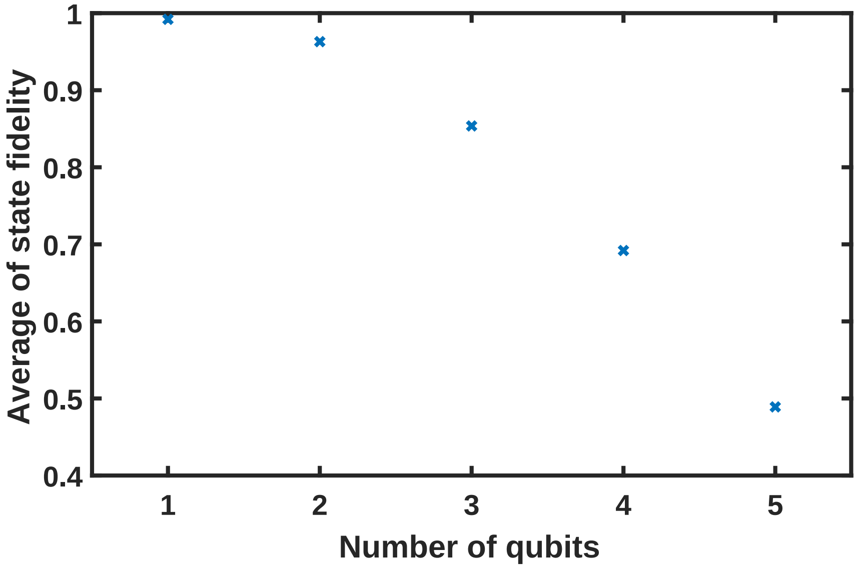


FIG. S11. Average of the state fidelity between the experimental and simulated results. For the five-qubit state, we partial trace different number of qubits and obtain the state fidelity of different qubit numbers. The average fidelity decays with the number of qubits.
